# Head-to-Head Comparison of Soluble vs. Qβ VLP Circumsporozoite Protein Vaccines Reveals Selective Enhancement of NANP Repeat Responses

**DOI:** 10.1371/journal.pone.0142035

**Published:** 2015-11-16

**Authors:** Farhat Khan, Mike Porter, Robert Schwenk, Margot DeBot, Philippe Saudan, Sheetij Dutta

**Affiliations:** 1 Structural Vaccinology Laboratory, Malaria Vaccine Branch, Walter Reed Army Institute of Research, Silver Spring, MD 20910, United States of America; 2 Cytos Biotechnology, Wagistrasse 25, 8952 Schlieren, Switzerland; London School of Hygiene and Tropical Medicine, UNITED KINGDOM

## Abstract

Circumsporozoite protein (CSP) of *Plasmodium falciparum* is a promising malaria vaccine target. RTS,S, the most advanced malaria vaccine candidate consists of the central NANP repeat and carboxy-terminal region of CSP displayed on a hepatitis B virus-like particle (VLP). To build upon the success of RTS,S, we produced a near full-length *Plasmodium falciparum* CSP that also includes the conserved amino-terminal region of CSP. We recently showed that this soluble CSP, combined with a synthetic Toll-like-receptor-4 (TLR4) agonist in stable oil-in-water emulsion (GLA/SE), induces a potent and protective immune response in mice against transgenic parasite challenge. Here we have investigated whether the immunogenicity of soluble CSP could be further augmented by presentation on a VLP. Bacteriophage Qβ VLPs can be readily produced in *E*.*coli*, they have a diameter of 25 nm and contain packaged *E*. *coli* RNA which serves as a built in adjuvant through the activation of TLR7/8. CSP was chemically conjugated to Qβ and the CSP-Qβ vaccine immunogenicity and efficacy were compared to adjuvanted soluble CSP in the C57Bl/6 mouse model. When formulated with adjuvants lacking a TLR4 agonist (Alum, SE and Montanide) the Qβ-CSP induced higher anti-NANP repeat titers, higher levels of cytophilic IgG2b/c antibodies and a trend towards higher protection against transgenic parasite challenge as compared to soluble CSP formulated in the same adjuvant. The VLP and soluble CSP immunogenicity difference was most pronounced at low antigen dose, and within the CSP molecule, the titers against the NANP repeats were preferentially enhanced by Qβ presentation. While a TLR4 agonist enhanced the immunogenicity of soluble CSP to levels comparable to the VLP vaccine, the TLR4 agonist did not further improve the immunogenicity of the Qβ-CSP vaccine. The data presented here pave the way for further improvement in the Qβ conjugation chemistry and evaluation of both the Qβ-CSP and soluble CSP vaccines in the non-human primate model.

## Introduction

Despite global eradication efforts, WHO estimates that more than 600,000 deaths per year can be attributed to malaria caused by *Plasmodium falciparum*. During a blood meal, a malaria-infected mosquito injects 10–200 live sporozoites into the host [1]. Each sporozoite that reaches and infects a liver hepatocyte can differentiate into as many as 30,000 merozoites which enter the bloodstream and infect red blood cells. Further replication of the blood stages lead to an exponential increase in parasite numbers. Hence a pre-erythrocytic stage vaccine would represent the best line of defense in preventing clinical manifestations of the disease [2]. While recent studies suggest that whole sporozoite vaccines elicit a strong protective immune response [3], the mass production and distribution of such products remains impractical. The development of a potent recombinant subunit vaccine along with the use of antimalarial drugs and vector control will be required to control and eradicate the disease [4].

Unlike pathogens where infection induces life-long immunity, the host immune response against malaria is weak, transient and non-sterilizing; mildly symptomatic carriers serve as a reservoir for continuous transmission [5]. The parasite evades the immune system primarily by remaining intracellular for the majority of its life-cycle and many of its polymorphic invasion antigens are actively secreted into the host serum after invasion. These secreted antigens are poor immunogens and may even be tolerogenic. Strategies that enhance the immunogenicity of malaria proteins are therefore of considerable interest for malaria vaccine development.

Amongst other characteristics, antigen size has been purported to be an important determinant of immunogenicity as soluble proteins (typically less than 10 nm) are poorly immunogenic [6]. Self-aggregation is a way of enhancing antigen size and is commonly achieved by emulsification in oil based adjuvants like Montanide [7], passive adsorption onto salts of trivalent aluminum [8] or covalent cross-linking using glutaraldehyde [9]. Alternatively, the size of an antigen can also be enhanced by conjugating to unrelated T cell epitope-rich soluble protein or particulate carrier e.g. Tetanus [10], KLH [11], PLGA [12] or a nanoparticle [13–16]. Virus-like particles (VLP) offer an advantage as they contain highly repetitive tertiary structures that are recognized as pathogen associated molecular patterns by the immune system [17–19]. VLPs range in size between 20–200 nm which is optimal for uptake and rapid transit to the lymphoid organs where they can interact with B and other antigen-presenting cells [6,20,21]. Therefore it is not surprising that the majority of successfully marketed recombinant protein vaccines are in fact VLPs (e.g. Gardisil and Cervarix against HPV and Engerix-B against Hepatitis). The most promising recombinant malaria vaccine candidate, RTS,S is also a VLP developed by fusing the most abundant sporozoite surface antigen circumsporozoite protein (CSP) with the human Hepatitis B virus envelope protein [22,23].

Plasmodia CSPs can be divided into three main regions: a conserved N-terminal region, followed by a NANP-NVDP repeat region and a cysteine rich C-terminal region. RTS,S contains only the *P*. *falciparum* CSP NANP repeats and the C-terminal region although several T helper and CTL epitopes have been mapped to the N-terminal region [24–26]. Furthermore the role played by the N-terminus in the process of sporozoite invasion [27] [28] suggests that immunity to the N-terminal region could enhance protection. We have previously manufactured an almost full-length *P*. *falciparum* CSP in *E*. *coli* consisting 19 NANP and 3 NVDP repeats and the majority of the N- and C-terminal regions (residues 26_Tyr_-127_Asp_ linked to 207_Pro_-383_Ser_) [29]. This soluble CSP antigen has been shown to be highly immunogenic and protective against transgenic parasite challenge in mice when adjuvanted with a stable emulsion (SE) based adjuvant that contains the potent Toll-like receptor-4 (TLR4) agonist Glucopyranosyl lipid A (GLA/SE) [29]. GLA/SE has been safely used in several human trials and is proposed as a potential adjuvant for CSP [30].

Here we wanted to test whether immunogenicity of soluble CSP could be further improved by conjugation to VLPs. To this end the efficacy of different formulations of soluble or VLP conjugated CSP were compared in mice. For these studies, a clinically validated VLP platform based on the Qβ capsid protein was used as an immunogenic carrier. Qβ-VLPs are composed of 180 monomeric capsid protein subunits of 14 kDa each [6] that can readily be produced in *E*.*coli*. Upon expression the monomers of Qβ self-assemble into a 25 nm icosahedral capsid within the *E*. *coli* cytosol. During expression, *E*.*coli* RNA gets packaged inside the Qβ particles and it serves as a potent agonist for TLR7/8 [31,32]. Since the recombinant Qβ does not contain the bacteriophage genetic information they are not infectious and cannot replicate. Qβ has also been used safely in multiple human trials targeting self- (angiotensin II, TNFα, ghrelin, IL-1β) as well as foreign antigens (globular domain of HA, Nicotine) [33–36]. We report here that Qβ VLP can be used as a carrier to improve the immunogenicity of CSP, in particular the NANP repeat epitope. The Qβ-CSP conjugate vaccine induced a potent and protective immune response against challenge with a transgenic rodent parasite expressing *P*. *falciparum* CSP gene. These data pave way for future non-human primate studies with the Qβ-CSP based vaccine formulations.

## Materials and Methods

### Production of the CSP-Qβ vaccine

Conjugation of CSP with Qβ was carried out according to the method described by Schmitz *et al*. [37] with some modifications as described below. Four milligrams of purified CSP (purity >99%; endotoxin <0.2 EU/μg protein) diluted in 1 ml PBS containing 3.5 M urea, 5 mM EDTA (pH 7.2) was mixed with a 15 molar excess of SATA (N-Succinimidyl S-Acetylthioacetate) in DMSO. The reaction mixture was incubated for 1 h at RT with slow stirring in a glass vial. Unreacted SATA was removed by passing through a PD-10 de-salting column equilibrated in 100 mM Sodium phosphate, 150 mM NaCl, 25 mM EDTA and 3.5 M urea (pH 7.2). Peak protein fraction was concentrated by Centricon (EMD Millipore). The SATA derivatized CSP was next treated with of 100 μl of 100 mM sodium phosphate, 150 mM NaCl, 25 mM EDTA, 0.5 M hydroxylamine and 3.5 M urea (pH 6.5) for 2 h at RT with slow stirring to generate the free–SH groups by deacetylation. The product was desalted again using a PD10 column equilibrated with 100 mM sodium phosphate buffer, 150 mM NaCl, 5 mM EDTA, 3.5 M urea (pH 6.5). Peak protein fractions were pooled and stored overnight at 4°C. GMP grade Qβ VLPs (protein purity >99%; RNA 0.3 μg/μg protein; endotoxin 0.004 EU/μg protein) in PBS were obtained from Cytos Biotechnology, Switzerland. First the Qβ (1.5 ml of 3 mg/ml) was treated with 100 μl of 3.8 mg/ml SMPH (Succinimidyl-1-6-[(β-maleimidopropionamido)hexanoate]) in DMSO. The reaction mixture was incubated in a glass vial at RT with slow stirring for 1 h. During this reaction the N-hydroxysuccinimide ester groups react to the primary amine groups of Qβ VLP, generating free maleimide groups on the Qβ. This reaction mixture was desalted using PD10 column, pre-equilibrated in 100 mM sodium phosphate, 150 mM NaCl and 5 mM EDTA (pH 6.5). Peak protein fractions were pooled. The CSP containing the free–SH groups was mixed in equal amounts with free maleimide group-containing Qβ and incubated at RT for 1 h with slow mixing. After 1 h, cysteine was added to the reaction to a final concentration of 1 mM and incubated at RT for 1 h to block any remaining free SH groups. A final buffer exchange was conducted by passing the conjugated product through a PD10 column. Peak protein fraction in PBS was pooled and stored at -70°C until further use.

### Product characterization

Coupling density of Qβ-CSP product was determined by densitometric analysis of a 4–20% gradient polyacrylamide gel. The cumulative band intensity of Qβ-CSP band and the free Qβ monomer band was determined and these were divided by the respective molecular weights. Then assuming that 180 Qβ monomers were present per particle [38], the average coupling density (number of CSP molecules per Qβ particle) was determined for 9 independent coupling reactions. Efficiency of coupling was defined as the percentage of the starting CSP amount (in mg), present as Qβ-bound or free form, in the final vaccine preparation. For all vaccinations the CSP dose was calculated by combining the amount of residual free CSP and CSP conjugated to Qβ. The hydrodynamic diameter of Qβ-CSP conjugate was determined using dynamic light scatter method on a Zetasizer Nano S (Malvern Instruments) in PBS pH 7.4 at 25°C. The endotoxin content of the final product was 0.15 EU/μg protein.

### Animals

Six- to eight-week old female C57Bl/6 (H-2^b^) mice were purchased from the Jackson Laboratories (Bar Harbor, ME). Animal procedures were conducted in compliance with the Animal Welfare Act and other federal statutes and regulations relating to animals and experiments involving animals and adhere to principles stated in the *Guide for the Care and Use of Laboratory Animals*, NRC Publication, 2011 edition. All procedures were reviewed and approved by the Walter Reed Army Institute of Research’s Animal Care and Use Committee (Protocol number:11-MVD-15), and performed in a facility accredited by the Association for Assessment and Accreditation of Laboratory Animal Care International. Humane euthanasia was performed by carbon dioxide displacement. Compressed gas was supplied to the chamber using a pressure-reducing regulator and a flow meter to maintain a displacement rate of ~20% of the chamber volume/min, this procedure was followed by cervical dislocation.

### Formulation and immunization with CSP and Qβ-CSP

The CSP in the Qβ-CSP vaccine was quantified as the sum of conjugated and unconjugated CSP. The total CSP amount was kept constant between vaccine groups within each experiment. Two or three doses of the vaccine (specific details in the results section) were administered at 3 weeks interval. For Montanide ISA720 adjuvant (Seppic, France) the antigen: adjuvant ratio was 3:7. The formulation was emulsified by vigorous vortexing for 10–15 min and 100 μl vaccine was administered intra-peritoneally. For GLA/SE (IDRI, Seattle WA) the antigen to adjuvant ratio was 1:1 v/v. No more than 100 μl vaccine containing 5 μg of GLA per dose was administered subcutaneously into the inguinal region. For Alum, 2% Alhydrogel (Invivogen, San Diego, California) was mixed in a 1:1 ratio by continuously inverting for 5 min and 100 μl vaccine was administered into the inguinal region. Mice were bled 3 weeks after the each immunization except for a bleed collected two weeks after the last dose and prior to challange. All mice were challenged with 3,000 transgenic sporozoites 2 weeks after the last vaccine dose, essentially as described previously [39,40].

### ELISA

Microtiter plates (Immulon 2HB) were coated overnight at 4°C with either the full-length protein (FL, 50 ng/well) or the repeat peptide (NANP, 20 ng/well) and washed 3 times with PBS + 0.05% Tween-20 (PBS/T). Thes-e plates were then blocked with PBS +1% Casein (PBS/C) for 1 h and washed 3 times with PBS/T. Sera were diluted 1:5,000 in (PBS/C) and serially diluted two-fold down each column of the plate in duplicates. Plates were incubated for 2 h at 22°C and washed 3 times with PBS/T. Fifty μl of 1:15000 diluted horse radish peroxidase conjugated anti-mouse IgG (Southern Biotech, Birmingham, AL) in PBS/C were added per well. After 1 h incubation at 22°C, plates were washed 4 times with PBS/T and developed by the addition of 50 μl/well ABTS peroxidase substrate system (KPL, Gaithersburg, MD) for 1 h at 22°C. The reaction was stopped by adding 50 μl of 5% SDS for 5 min, and the absorbance at 415 nm was measured using a microplate reader (Synergy 4, Biotek, Highland Park, VT). The antibody titer was calculated as the serum dilution that produced an absorbance of 1.0 optical density (O.D.) units using Gen5^TM^ software (Biotek).

### Isolation of sporozoites for challenge

A transgenic *P*. *berghei* parasite expressing a functional copy of full-length *P*. *falciparum* CSP gene was used for challenge studies [39,40]. Fresh naïve mouse serum was used to supplement RPMI to a final concentration of 5% v/v. Sporozoites were collected using the Ozaki method and counted on a hemocytometer [41]. Sporozoites were diluted to 25,000 per ml in RPMI/serum and 100 μl of this suspension was injected into the lateral tail vein.

### Monitoring mouse infection

Levels of parasitemia in the mice were monitored daily using thin blood smears beginning at day 5 post-challenge. Blood smears were methanol fixed and stained with Giemsa. Mice found to be infected with blood stages of the parasite were sacrificed and recorded as “not protected” while mice that did not develop blood stage parasitemia up to 14 days post-challenge were sacrificed and reported as protected.

### Immunofluorescence assay (IFA)

Sporozoites of *P*. *falciparum* NF54 strain were air dried on a glass slide and stored in the freezer. Slides were thawed for 15 min at RT, fixed in methanol and blocked with 1% BSA for 30 min at RT. Diluted serum samples (primary antibody) in 1% BSA were then added to each well (15 μL) for 1 h. The slide was washed with PBS for 2 min and air dried. 15 μL of 1:5,000 FITC-conjugated secondary antibody was next added and incubated at RT for 1 h. Slide was washed 3 times and a coverslip was mounted on the well using Vectashield^TM^ hard mount medium (Vector Labs, Burlingame, CA). Slides were read using a fluorescent microscope.

### Luminex

Luminex beads were purchased from Luminex Corporation (Austin, TX) and 96-well Luminex Multi-screen BV 1.2 micron assay plates were acquired from Millipore Corporation (Billerica, MA). NANP peptide (0.5 μg) or a recombinant protein representing the C-terminal (C-term) region of CSP (0.4 μg) were coupled to 5x10^6^ beads according to the manufacturer’s instructions. Representative serum samples (high and low responders) were tested at a series of dilutions. A titration curve was drawn and a linear range dilution was chosen. All serum samples were run at this one selected dilution. Typically sera were diluted 1/1000 in PBS containing 1% BSA (assay buffer). Fifty μl of the sample was added to the wells along with 50 μl of assay buffer containing 3,000 beads coated with NANP and 3000 C-term protein coated beads (with two different bead signatures). The plates were agitated on a shaker for 1 h at RT and washed in assay buffer containing 0.05% Tween-20. 100 μl of assay buffer containing PE-labeled-(mouse IgG subclass)-specific antibody (Jackson Immunoresearch; West Grove, PA) was then added to the wells and the plate was agitated for an additional hour. The plate was washed and median fluorescence intensity (MFI) was measured for ~100 beads per well using the Luminex 200 system (Luminex Corporation, Austin, TX).

### Statistical analysis

ELISA and Luminex titers were log transformed and two-way comparisons were made by unpaired t-test on the GraphPad Prism software (La Jolla, CA). Multiple comparisons were made by ANOVA and p values were corrected using Tukey’s method. Statistically significant difference in group means was indicated as **** (p<0.0001), *** (p<0.001), ** (p<0.01), or * (p<0.05). Protection results in each group were compared to the control group and P values for Fischer’s exact test were reported where significant. Grouped protection data was analyzed by Chi square test. All graphs show individual data points and the mean ± SEM.

## Results

### Chemical coupling of CSP to Qβ

**Fig 1:**
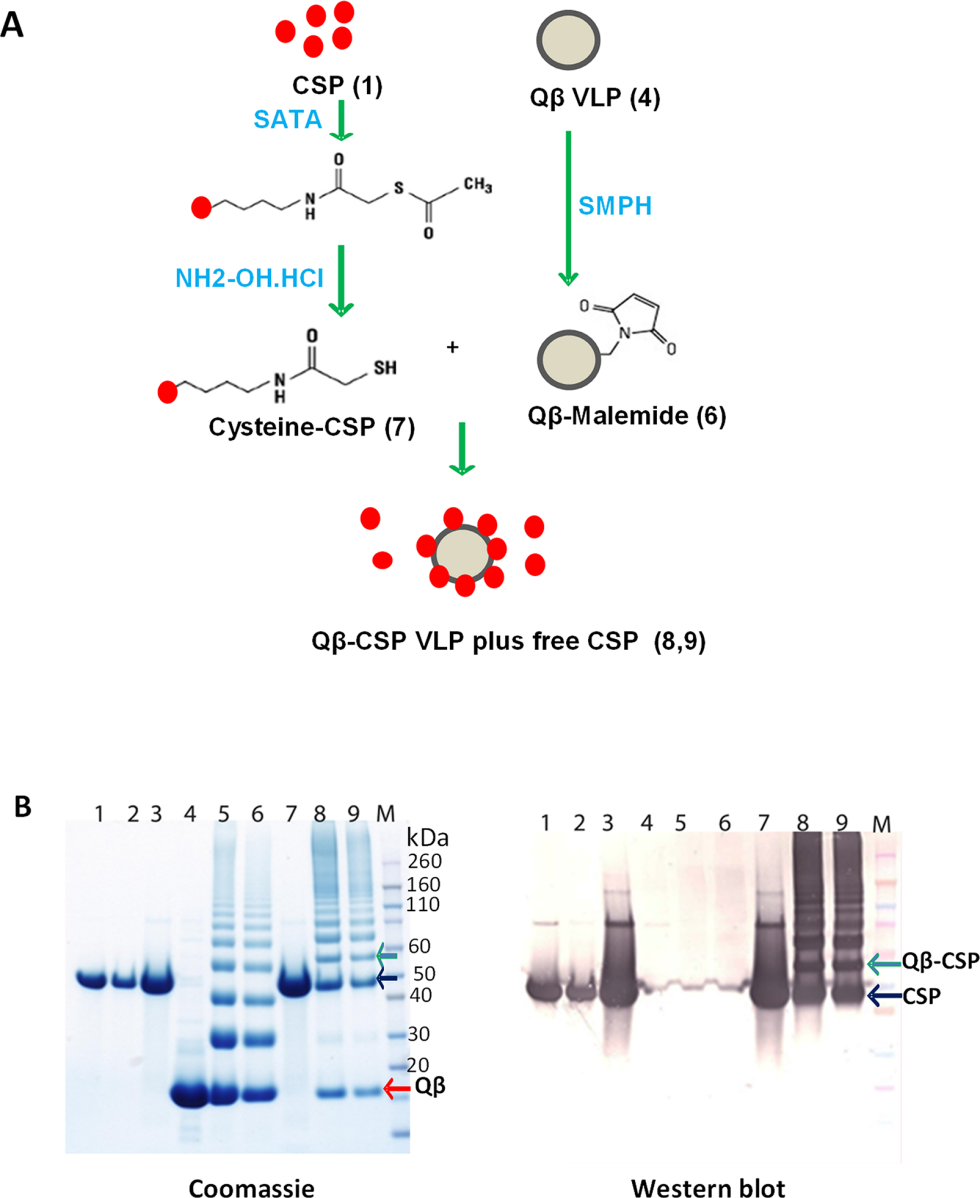
Production of the Qβ-CSP vaccine. A, Outline of the conjugation process. Numbers in parentheses correspond to the respective lane number in Fig 1B. B, CSP and Qβ proteins analyzed by reducing SDS-PAGE followed by coomassie blue staining (left) or western blot using anti-CSP polyclonal mouse antibodies (right). Lane 1, soluble CSP protein; 2, CSP diluted in urea; 3, SATA treated and desalted CSP; 4, Qβ protein; 5, SMPH treated Qβ; 6, desalted Qβ-Malemide; 7, deacetylated and desalted CSP; 8, CSP-Qβ conjugate; 9, desalted CSP-Qβ conjugate (final vaccine); M, molecular weight marker.

CSP was coupled to the Qβ particle using a multi-step process (Fig 1A) and proteins at each conjugation step were analyzed by reducing SDS-PAGE (Fig 1B). Dilution in urea helped minimize protein loss (Fig 1B, lane 2) while sulfhydryl residues were added to CSP (lane 3). Purified Qβ protein (lane 4) was treated with SMPH to add the malemide group(s) (lane 5). The added sulfhydryl group(s) on CSP was de-protected (lane 7) and mixed with malemide derivatized Qβ to result in Qβ-CSP conjugate vaccine (lane 8, 9). Soluble CSP and Qβ monomer migrated at ~45 kDa (black arrow) and ~14 kDa (red arrow) respectively (Fig 1B). The addition of sulfhydryl groups did not cause a shift in SDS-PAGE profile of CSP but malemide addition to Qβ resulted in higher molecular weight bands that corresponded to 2x, 3x, 4x and 5x Qβ multimers (Fig 1B, lanes 5, 6). Covalent linkage of CSP to a Qβ monomer resulted in a ~58 kDa Qβ-CSP band (Fig 1B, lanes 8, 9). The higher molecular weight bands in conjugate lane corresponded to the cross-linking of a CSP monomer to 2x, 3x, 4x and 5x Qβ multimers. During the conjugation process CSP was followed by western blotting with polyclonal anti-CSP mouse antibody (Fig 1B, right panel).

To determine the ratio of the Qβ-CSP conjugate band *vs*. the free Qβ monomer species coomassie stained gels were analyzed by densitometry. After adjusting for band intensity changes due to the molecular weight difference and assuming 180 Qβ monomers per particle [38] the average coupling density of the conjugation process was found to be between 12–25% for 9 independent coupling reactions. This indicates that an average of ~30 CSP molecules were conjugated to each Qβ VLP. Efficiency of CSP coupling was relatively low with ~40–50% of the starting CSP binding to Qβ and ~3–10% remained free in the final vaccine preparation. For all vaccinations the CSP dose was calculated by combining the residual free and conjugated CSP.

### Identity, immunological activity and stability

**Fig 2:**
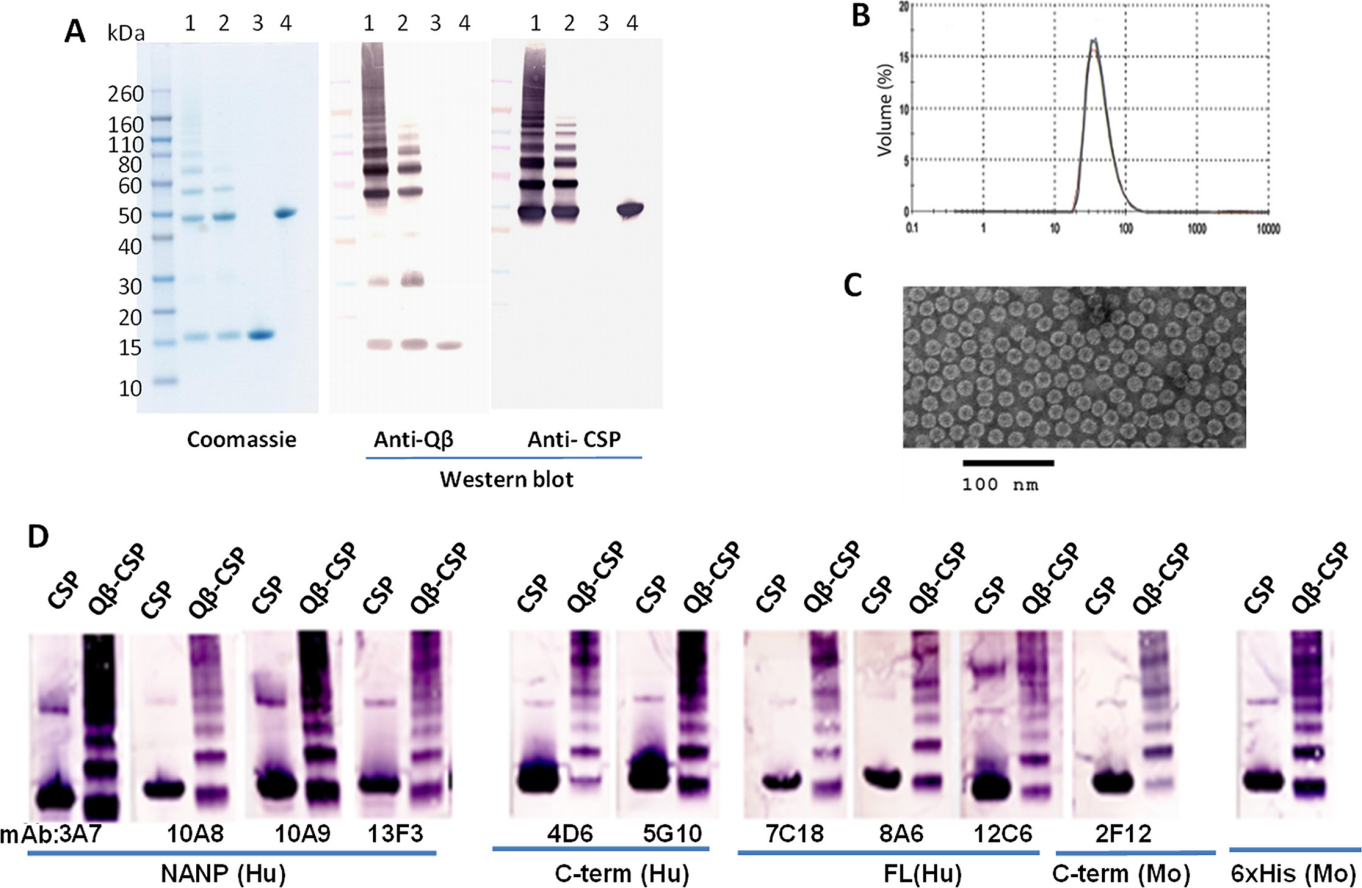
Characterization of Qβ-CSP vaccine. A, identity of the product bands was confirmed using reducing coomassie blue staining and western blot with polyclonal mouse anti-Qβ and anti-CSP antibodies. Lanes 1, 2, Qβ-CSP; lane 3, unconjugated Qβ; lane 4, unconjugated CSP. B, particle size distribution of Qβ-CSP by dynamic light scatter analysis. C, electron micrograph of the negatively stained Qβ-CSP. D, immunological reactivity of the soluble CSP and Qβ-CSP against CSP-specific mAbs targeting the NANP repeats, the C-term region or epitopes present only on full-length (FL) CSP. Mabs labelled as “Hu” were produced in humanized mice and “Mo” were produced in wild-type mice.

Covalent conjugation of CSP and Qβ was confirmed using reducing western blots with anti-Qβ and anti-CSP antibodies (Fig 2A). Lanes 1 and 2 correspond to the conjugated Qβ-CSP product while lanes 3 and 4 were loaded with unconjugated Qβ and CSP respectively. All high molecular weight bands in Qβ-CSP (lanes 1 and 2) but not free CSP or free Qβ (lanes 3 and 4) showed dual reactivity with anti-Qβ and anti-CSP antibodies (Fig 2A). Free Qβ monomer and dimer bands at ~15 and ~30 kDa reacted only with anti-Qβ antibody (Fig 2A, lanes 1, 2, 3). This data confirmed the covalent conjugation of CSP with Qβ.

Hydrodynamic diameter of Qβ-CSP formulation deduced by dynamic light scatter revealed that 95% of the product had an average diameter of ~25 nm (Fig 2B). An electron micrographic examination of negatively stained Qβ-CSP confirmed these sizing results (Fig 2C). The western blot of soluble CSP showed positive reaction with CSP-specific monoclonal antibodies (mAbs) targeting the repeat region (3A7, 10A8, 10A9, 13F3), the C-terminal region (4D6, 5G10, 2F12) and mAbs recognizing only the full-length CSP (7C18, 8A6, 12C6) (Fig 2D). All of the Qβ-CSP bands also reacted to these mAbs indicating that the conjugate had a similar epitope range as soluble CSP. Stability of CSP and Qβ-CSP was deduced by SDS-PAGE and both were stable at -80° and 4°C during a 7 day stability test (data not shown).

### CSP *vs*. Qβ-CSP in Montanide

**Fig 3:**
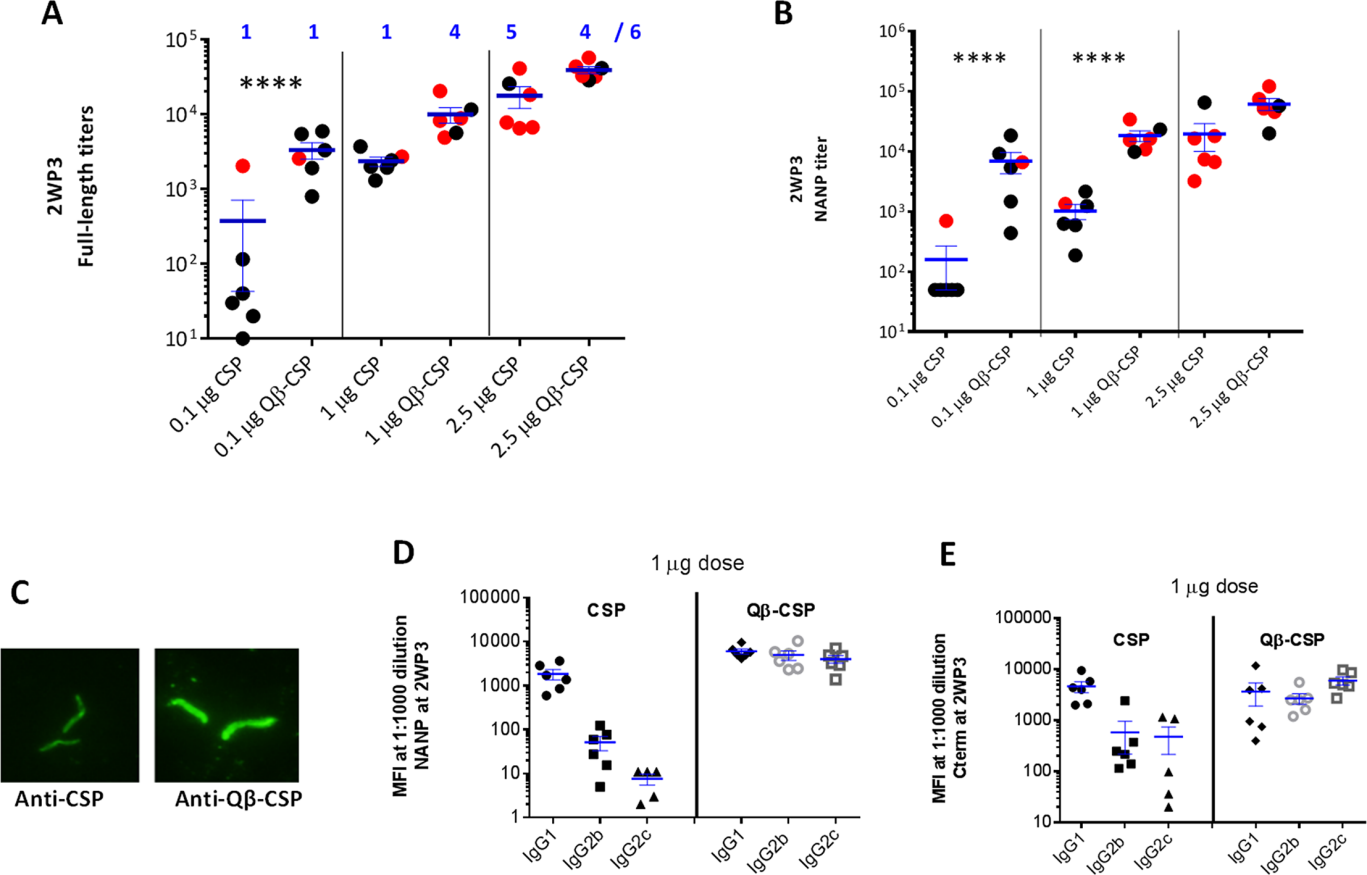
Qβ-CSP *vs*. CSP in Montanide. Groups of 6 mice were vaccinated thrice with 2.5, 1 and 0.1 μg CSP or Qβ-CSP in Montanide. A, B show the individual data points and mean±SEM titers against full-length protein (A) and NANP repeat peptide (B) 2 weeks post 3^rd^ vaccination (2WP3). **** (p<0.0001 for ANOVA followed by Tukey’s multiple comparisons test); red data points correspond to mice protected 14 days post challenge and numbers (blue) were protected out of 6. C, Immunofluorescence image of methanol fixed sporozoites stained with 1:2500 dilution of anti-CSP pool (left) or Qβ-CSP serum pool (right) for the 1 μg dose groups. D, E show IgG1, IgG2b and IgG2c levels measured by Luminex and expressed as median fluorescence intensity (MFI) at 1:1000 serum dilution against the NANP peptide (D) or the C-term protein (E).

Groups of six mice were vaccinated three times with Montanide (Mont) adjuvanted PBS (ctrl) or 0.1, 1 and 2.5 µg CSP and Qβ-CSP vaccines at 3 week intervals (Fig 3). The antigen dose indicated was of total CSP (free + Qβ conjugated) in each vaccine formulation. The mean titer of all vaccine groups increased following two booster vaccinations and the maximal difference between CSP and Qβ-CSP was observed at the lowest antigen dose, in particular for the NANP titers (S1 Fig). Two weeks post third vaccination, the mean CSP and NANP peptide-specific antibody titers increased with escalating antigen dose suggesting that this dose range was not saturating in C57Bl/6 mice. In the 2.5 μg high dose group, the full-length and NANP titers of Qβ-CSP group were higher than CSP but this difference was not significant. At 1 μg dose, the mean titers against full-length antigen were 6 fold higher for the Qβ-CSP group, but this difference was also not significant. In contrast, the NANP titers of the Qβ-CSP group were significantly higher as compared to soluble CSP at the 1 μg dose. At the 0.1 μg dose both the Qβ-CSP-induced CSP and NANP specific titers were significantly higher than the CSP-induced titers (Fig 3A and 3B). Remarkably the mean full-length and NANP titers of mice receiving 0.1 μg Qβ-CSP were similar to those induced by a 10 fold higher 1 μg of soluble CSP. Antibodies against both vaccines recognized the native CSP on fixed sporozoite surface in an immunofluorescence assay (Fig 3C). At 1:2500, the Qβ-CSP pool (1 μg dose) showed a higher intensity staining than the CSP pool.

Analysis of NANP and C-term specific antibody subclasses for the 1 μg dose groups showed that Qβ-CSP induced a higher magnitude of NANP-specific IgG1, IgG2a, IgG2c (unpaired T test, all P values <0.05) and C-term-specific IgG2b, IgG2c (P values <0.05). Qβ-CSP induced a more balanced IgG1:IgG2b or IgG2c, while CSP induced primarily IgG1 subclass with little or no IgG2 antibodies against either the C-term or the NANP repeat (Fig 3D and 3E). At two weeks post third dose, vaccinated mice along with 6 naïve control mice were challenged with a transgenic *P*. *berghei* strain that carries the full-length *P*. *falciparum* CSP gene [40]. All 6 PBS control mice were infected by day 6 and in accordance with the observed antibody titers, no difference between vaccines was observed at the highest dose with 4/6 and 5/6 mice protected in Qβ-CSP and CSP groups respectively (Fig 3A and 3B; red symbols). At the 1 μg antigen dose higher protection was observed in the Qβ-CSP conjugate group as compared to CSP (4/6 *vs*. 1/6). Hardly any protection was observed at the lowest dose, 0.1 μg CSP (1 of 6 mice protected in both groups).

The protection experiment in Montanide at the 1 μg CSP antigen dose was repeated using a 2-dose schedule (S2 Fig). In this repeat experiment, the higher immunogenicity of Qβ-CSP was confirmed (S2A and S2B Fig) with significantly higher full-length and NANP specific titers. The titer of the Qβ-CSP-induced antibodies remained higher the CSP group over the next 52 week follow-up period (S2C Fig). A balanced IgG1:IgG2b or IgG2c response induced by the Qβ-CSP+Mont vaccine was also confirmed (S2D and S2E Fig, no significant difference between IgG1, IgG2a and IgG2c). Upon challenge all the PBS control mice became infected and no protection was observed following two doses of 1 μg CSP+Mont. In contrast, 4/10 Qβ-CSP group mice were protected (Fisher’s test P = 0.01). These experiments confirmed that Qβ-CSP vaccine was more immunogenic and protective than soluble CSP. However, a higher antigen dose may minimize the immunogenicity gap between soluble and particulate vaccine in this mouse model. Particulate presentation had a higher impact on NANP titers as compared to the full-length antigen titers.

### CSP *vs*. Qβ-CSP in Alum

**Fig 4:**
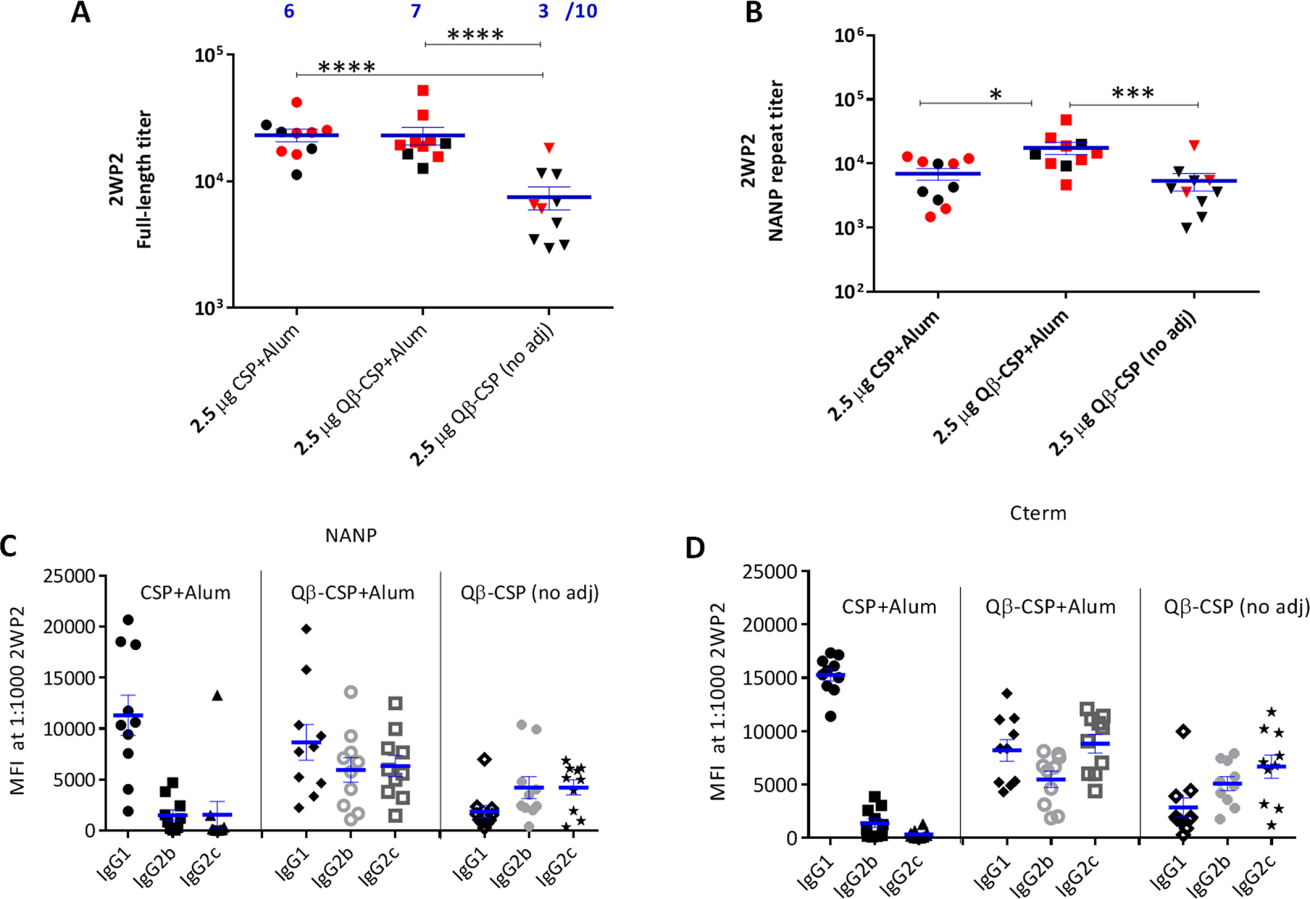
Qβ-CSP *vs*. CSP in Alum. Groups of 10 mice received 2 doses, 3 weeks apart of 2.5 μg CSP+Alum, Qβ-CSP+Alum or Qβ-CSP without an adjuvant. A, B show the mean±SEM of the full-length and NANP-specific ELISA titers at 2 weeks after the second vaccination. **** (p<0.0001); *** (p<0.001); * (p<0.05); red symbols represent protected mice and number (blue) protected out of 10. C, D, show the IgG1, IgG2b and IgG2c responses measured by Luminex and expressed as MFI at 1:1000 dilution against NANP peptide (C) or C-term protein (D).

Qβ-CSP and CSP were next compared in Alum, an adjuvant commonly used for human vaccines. Ten mice per group were given 2 doses of 2.5 μg CSP+Alum, Qβ-CSP+Alum or Qβ-CSP without an adjuvant (Fig 4). The non-adjuvanted Qβ-CSP induced significantly less full-length specific antibodies than adjuvanted CSP and Qβ-CSP groups. While Alum adjuvanted CSP and Qβ-CSP induced similar levels of full length-specific antibodies the NANP responses induced by Qβ-CSP+Alum were higher than CSP+Alum (Fig 4A and 4B). Remarkably, the NANP responses of the non-adjuvanted Qβ-CSP were similar to Alum adjuvanted soluble CSP. At the subclass level, CSP+Alum induced high IgG1 and little or no IgG2 against NANP or C-term (Fig 4C and 4D). In sharp contrast, Qβ-CSP (with or without Alum) induced a balanced IgG1:IgG2 response. Remarkably the NANP and C-term-specific IgG2c response of the CSP+Alum vaccine was lower than the non-adjuvanted Qβ-CSP group (both P values <0.001) providing direct evidence that conjugation to Qβ was sufficient to enhance IgG2 antibody responses. Mice were challenged at 2 weeks post second vaccination and unlike the two previous experiments, 1/10 mice in the control group (PBS+Alum) did not become infected. In the non-adjuvanted Qβ-CSP group 3/10 mice were protected while the CSP+Alum and Qβ-CSP+Alum vaccines showed similarly high protection rates with 6/10 and 7/10 mice protected respectively. When compared to the control group only the Qβ-CSP+Alum protection was statistically significant (Fig 4A, Fischer’s test P = 0.01). In this 2-dose experiment, Alum emerged as a highly potent adjuvant in mice not only for Qβ-CSP but also for soluble CSP vaccine and conjugation to Qβ was found to more selectively enhance the NANP titers and IgG2 responses of CSP.

### CSP *vs*. Qβ-CSP in SE, GLA/SE and Alum

**Fig 5:**
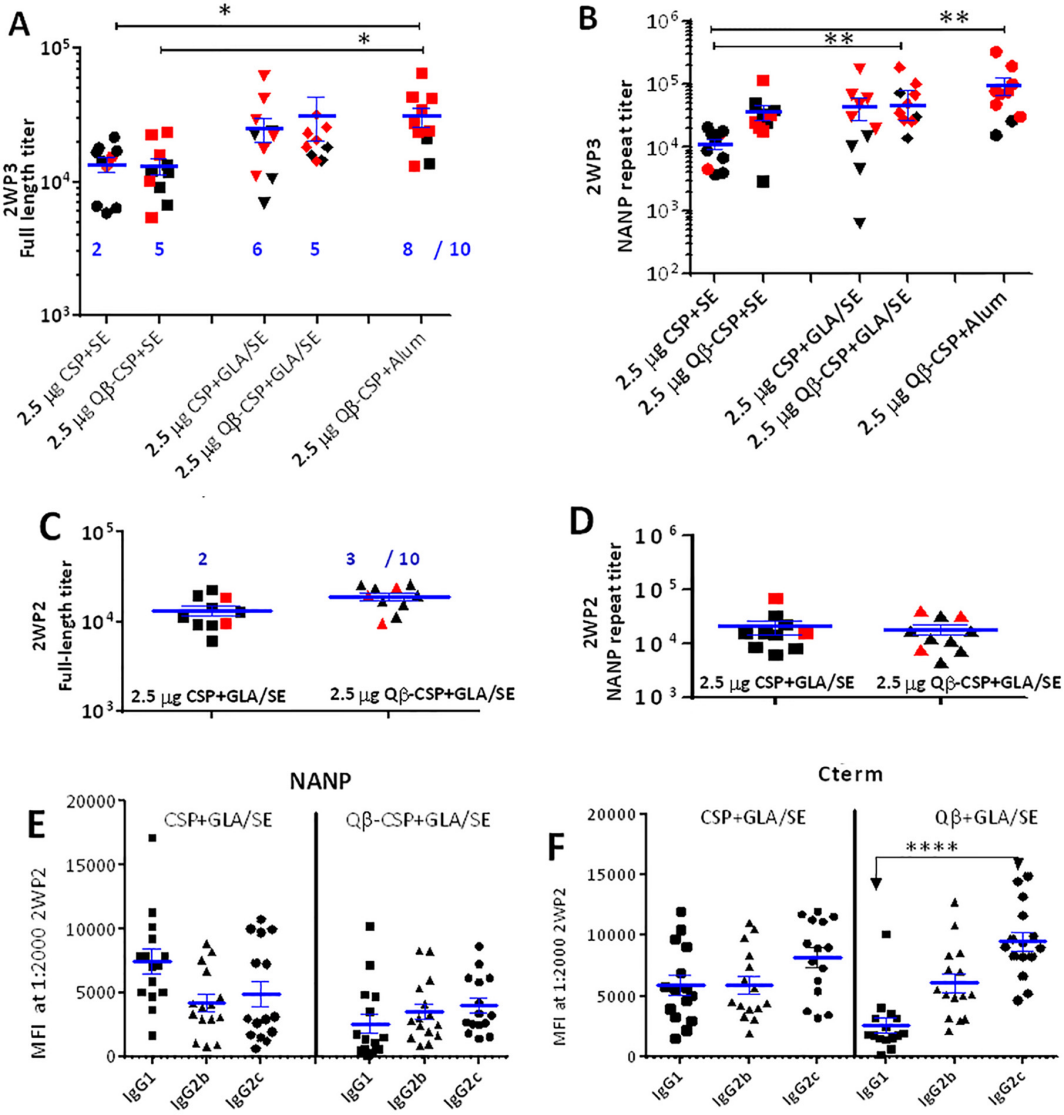
Qβ-CSP *vs*. CSP in SE, GLA/SE and Alum. Groups of 10 mice received 3 doses, 3 weeks apart of 2.5 μg CSP+SE, Qβ-CSP+SE, CSP+GLA/SE, Qβ-CSP+GLA/SE or Qβ-CSP+Alum. A, B show the mean±SEM of the full-length and NANP-specific ELISA titers at 2 weeks post third vaccination. ** (p<0.01); * (p<0.05); red symbols represent protected mice and number (blue) protected out of 10. C, D, E and F are data from an independent 2-dose study. 15 mice received 2 doses of 2.5 μg CSP+GLA/SE and Qβ-CSP+GLA/SE, three weeks apart. C, D show the mean±SEM of full-length and NANP-specific ELISA titer from the 10 challenged mice at 2 weeks after the second vaccination. Red symbols were protected mice and number represent protected out of 10 (blue). E, F, IgG1, IgG2b and IgG2c responses of 15 mice measured by Luminex and expressed as MFI at 1:2000 dilution against NANP peptide (E) or C-term protein (F).

We next compared Alum with another human-use adjuvant GLA/SE, a stable emulsion (SE) based adjuvant that contains a synthetic TLR4 agonist GLA. We previously showed potent antibody and protective responses were induced by CSP when formulated with GLA/SE in this transgenic parasite mouse challenge model [29]. Groups of 10 mice were immunized three times with 2.5 μg CSP+SE, Qβ-CSP+SE, CSP+GLA/SE, Qβ-CSP+GLA/SE or Qβ-CSP+Alum. At 2 weeks post third vaccination mice were challenged with transgenic sporozoites (Fig 5A and 5B). Similar full-length titers were observed between the two SE-adjuvanted groups but as observed with Alum, the NANP-specific titers of the Qβ-CSP group were ~3 fold higher than CSP although this difference was not statistically significant. No difference in immunogenicity was observed between CSP *vs*. Qβ-CSP in the GLA/SE. As observed in the previous Alum experiment, the Qβ-CSP+Alum group again showed high antibody titers that were not significantly different from GLA/SE adjuvanted CSP or Qβ-CSP groups (Fig 5A and 5B). Upon challenge all PBS control mice became infected while 5/10 Qβ-CSP+SE (P = 0.03, compared to the PBS group) *vs*. 2/10 CSP+SE (not significant when compared to the controls) group mice were protected. Similar protection was observed in the CSP+GLA/SE (6/10; P = 0.01) and Qβ-CSP+GLA/SE (5/10; P = 0.03) groups (Fig 5A and 5B). In accordance with the high antibody responses, the highest protection was observed in the Qβ-CSP+Alum group (8/10; P = 0.0007). Significantly better protection observed with Qβ-CSP+SE than with CSP+SE, despite similar full length titers suggest that NANP titers may be a more important correlate of protection than total CSP antibody titers. Taken together these results suggest that while soluble CSP immunogenicity was enhanced by TLR4 agonist addition, the Qβ-CSP vaccine induced high NANP titers and protection because of its particulate nature and the presence of the TLR7/8 agonist in its core.

Since no difference was observed between the GLA/SE adjuvanted CSP and Qβ-CSP in the above experiment, we repeated the study using a more restricted 2-dose schedule. Two groups of 15 mice received 2 immunizations of 2.5 μg CSP+GLA/SE or Qβ-CSP+GLA/SE at 3 wk interval (Fig 5C and 5D). Control mice were given PBS+SE. Even with the abbreviated vaccination schedule, no differences were noted in the immunogenicity of the GLA/SE adjuvanted CSP and Qβ-CSP (Fig 5C and 5D). At the subclass level, a balanced IgG1:IgG2 response against NANP and C-term was induced by CSP+GLA/SE, in contrast combining the Qβ-CSP with GLA/SE shifted the C-term responses further towards higher IgG2 subclass of antibodies compared to IgG1 (Fig 5E and 5F). Ten mice were challenged 2 weeks after the second dose (Fig 5C and 5D). All mice in the control PBS+SE group became infected; but in contrast to the previous 3-dose study only 2/10 and 3/10 mice were protected following 2 vaccinations with CSP and Qβ-CSP respectively (Fisher’s test, P values not significant). Hence the overall protection achieved with GLA/SE was inferior to Alum where more than half of the animals were protected with a 2-dose regimen (Fig 4). Importantly, the difference between CSP and Qβ-CSP vaccine immunogenicity was minimal when a TLR4 containing adjuvant GLA/SE was added to the two vaccines.

## Discussion

A potent antibody response is elicited when foreign epitopes are displayed as a repetitive mosaic on the surface of a particle [42]. Presentation on nanoparticles and VLPs is known to enhance the immunogenicity of *P*. *falciparum* CSP [43–45]. The majority of these particle platforms require CSP be expressed as a fusion protein, which poses steric and folding limitations on the size of the CSP construct. The Qβ conjugation technology allowed us to display a nearly full-length CSP that was highly purified and available as a GMP product [29]. Likewise, additional antigens with complex folding requirements can also be displayed on the Qβ platform because the particle and the antigen are separately folded and purified. Most importantly, this platform allowed us to make a head-to-head immunogenicity comparison of the same CSP antigen in its soluble and particulate form. In RTS,S, the CSP gene is directionally fused to the hepatitis B S-antigen and on average one copy of CSP is displayed for every four copies of hepatitis S-antigen monomers [46]. In the current study, every 6th Qβ monomer was linked to a CSP molecule, the orientation of these epitopes could not be controlled due to the nature of the linkage chemistry. Moreover, about half the starting CSP amount was lost during the conjugate production process. Improvements in coupling chemistry that allow directional conjugation of CSP to Qβ are needed to economize process yields and it may also lead to better cross-linking of the B-cell receptor, further improving the immunogenicity.

Despite disparities in adjuvants and experimental design a pattern of differences between CSP and Qβ-CSP immunogenicity emerged. While others have proposed particles as non-adjuvanted vaccines [15], our mouse data showed a clear immunological benefit of administering the Qβ-CSP particles in an adjuvant like Alum. Of all adjuvants tested, Alum-adjuvanted Qβ-CSP showed the best protective immune response in the C57Bl/6 mouse model. This makes a strong case for advancing Qβ-CSP formulated in Alum. While the results in mice were encouraging, many Alum-adjuvanted vaccines have failed to induce a potent protective response in past CSP human trials [46]. Further studies in non-human primates would therefore be required to confirm the excellent immunogenicity of Qβ-CSP+Alum formulation reported here in mice.

We have previously demonstrated that a synthetic TLR4 agonist lipopolysaccharide analog, GLA, significantly enhances soluble CSP immunogenicity and protective efficacy [29], however Qβ-CSP vaccine contained very low levels of bacterial endotoxin. The *E*. *coli* RNA present within the Qβ particles is a known TLR7/8 agonist and a Th1-driving immune modulator [31,32]. Not surprisingly all formulations of Qβ-CSP tested here induced high levels of cytophilic IgG2c antibodies that are indicative of Th1-biased immune response. Most importantly the improvement in immunogenicity vis-à-vis NANP repeat titer, protection and balanced IgG1:IgG2 response [29] was most pronounced when comparisons were made in adjuvants lacking an external TLR agonist (i.e. either Montanide, SE, or Alum alone). It is difficult to discern between the presence of TLR7/8 agonist and particulate presentation as the major cause of improved immunogenicity of CSP. And while higher immunogenicity of VLPs is well documented [47,48], present data suggests that a TLR agonist adjuvanted soluble CSP vaccine may still represent an effective and pragmatic approach towards developing a more cost-effective malaria vaccine [29]. No further improvement in immunogenicity was observed when Qβ-CSP was tested in GLA/SE. The apparent lack of synergy between TLR4 and TLR7/8 ligands was similar to that observed when mixture of GLA and R848 was previously tested with CSP [29]. In adjuvants where no TLR4 agonist was present the biggest difference between CSP and Qβ-CSP was observed at the lowest antigen dose (Fig 3A and 3B and S1 Fig). It is important to note, that the typical antigen dose tested in these mice (2.5 μg) was much higher than what could be administered on mg/kg basis to humans. Hence enhanced immunogenicity of Qβ at limiting antigen doses may be important vis-à-vis human responses.

**Fig 6:**
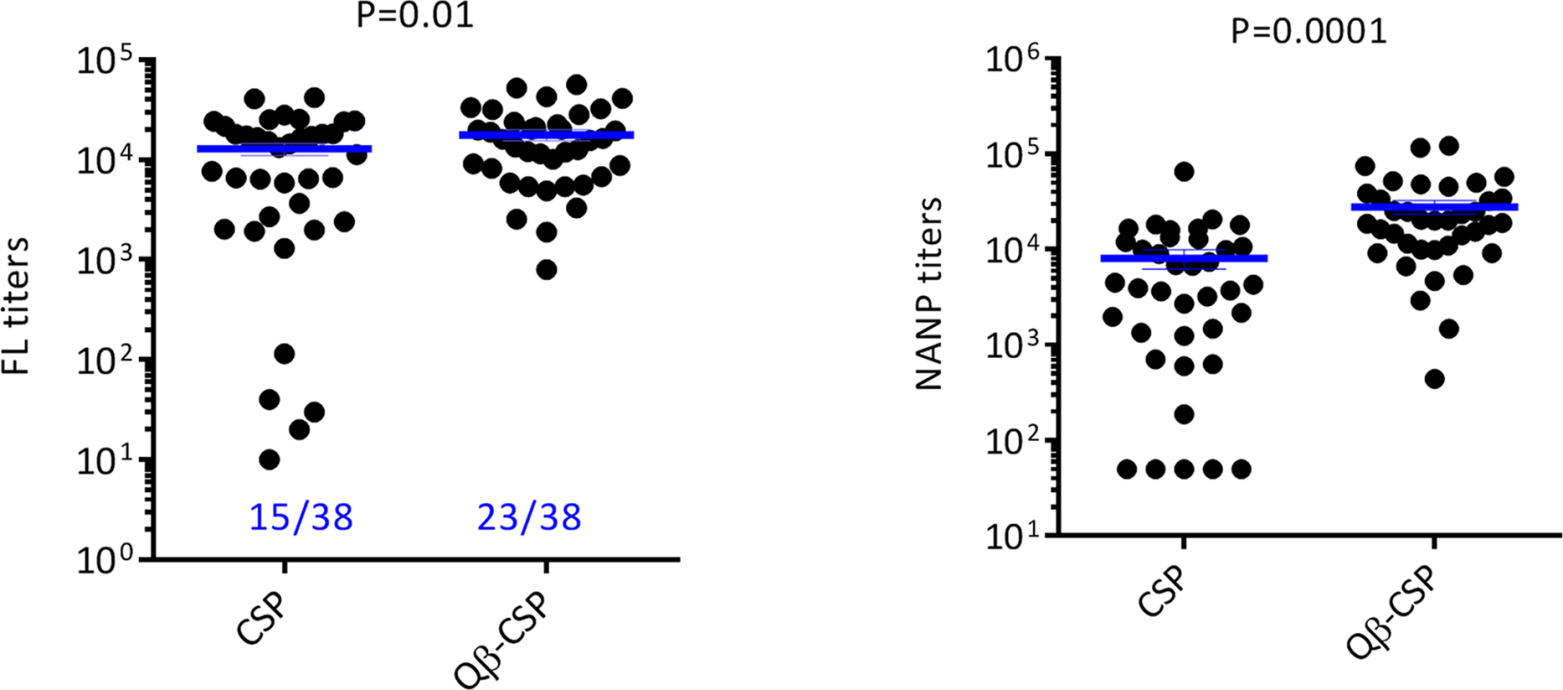
Combined ELISA titer and protection data of CSP *vs*. Qβ-CSP groups. Full-length (left) and NANP (right) titers were plotted for individual animals in Montanide, Alum and SE adjuvanted CSP and Qβ-CSP groups. Combined protection data is indicated (blue). Lines are mean±SEM and the P values are for unpaired T test performed on log transformed titers.

When we combined immunogenicity and protection data across experiments for groups where no external TLR4 agonist were present (Montanide, Alum and SE), the pre-challenge median and mean anti-full-length titer of Qβ-CSP group were 1.2 and 1.4 fold higher respectively, compared to the soluble CSP vaccine (P = 0.01). In contrast, the anti-NANP titers showed a much higher, 4.7 and 3.5 fold increase, in median and mean titers respectively, in favor of the Qβ-CSP vaccine (P = 0.0001; Fig 6). This selective increase in NANP responses translated into a marginal improvement in protection in the Qβ-CSP group however, this difference in protection was not statistically significant (60% vs 39%; Chi square test P = 0.06). Within CSP the titers against the NANP repeat region is believed to be strongly associated with protection in RTS,S challenge studies [49]. Moreover, repeatless CSP vaccines have failed to induce protection in humans [50] and in transgenic challenge models of malaria [40,45]. We have also recently shown that CSP specific IgG2 antibodies correlate with protection better than IgG1 [29] and similar observations are reported for protection against influenza [32]. Hence, the enhancement of repeat responses as well as IgG2 antibodies by Qβ presentation is an important observation. T cell-independent activation of B cells is well known for antigens displayed as a repetitive mosaic due to more efficient cross-linking of the B cell receptor [42]. NANP epitope is a known T-cell independent epitope [51] and particulate presentation along with the presence of the TLR7/8 agonist could be way to effectively enhance anti-NANP responses.

Soluble forms of many malaria vaccine candidates AMA1, MSP1 and Pfs25 in human-use adjuvants have thus far failed to induce protective immune response but when formulated in highly reactive adjuvants such as Freunds Complete and Montanide, potent anti-malarial responses have been reported [52–56]. While some vaccine candidates require high levels of antibodies, others like Rh5 may have naturally evolved to be weak immunogens [57]. The epitope specific enhancement of NANP responses suggests that particle display technologies may also help augment the immunogenicity of these sexual and asexual malaria vaccine candidates.

## Supporting Information

S1 Fig Mean ELISA titer against full-length and NANP in sera collected at 2 weeks post 1^st^, 2^nd^ or 3^rd^ vaccination (2wp1, 2wp2 and 2wp3, respectively). The data revealed maximum difference between CSP and Qβ-CSP immunogenicity was at the lowest antigen dose, particular for the NANP titers.
(TIF)

S2 Fig Groups of 15 mice received two doses of 2.5 μg Qβ-CSP+Mont or CSP+Mont at three week interval. A, B show the FL and NANP response 2 weeks after the second vaccination of 10 challenged mice. **** (p<0.0001); red symbols represent protected mice and numbers protected out of 10 (blue). C, Mean full-length ELISA titer of 5 mice followed for 1 year. D, E show IgG1, IgG2b and IgG2c responses measured by Luminex and expressed as MFI at 1:1000 dilution against NANP peptide (D) or C-term protein (E).
(TIF)
