# Supplementary figures and images for: Head-to-Head Comparison of Soluble vs. Qβ VLP Circumsporozoite Protein Vaccines Reveals Selective Enhancement of NANP Repeat Responses
Source: PLoS One. 2015 Nov 16;10(11):e0142035. doi: 10.1371/journal.pone.0142035 (PMC4646581; doi:10.1371/journal.pone.0142035)

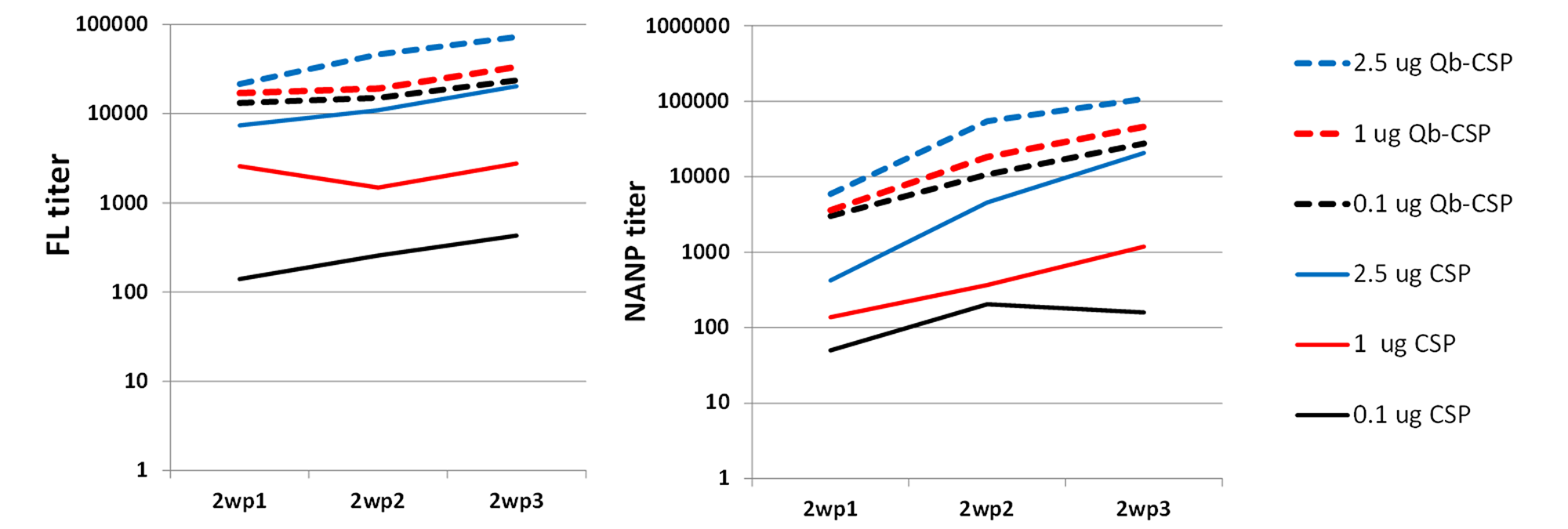

Supplement: S1 Fig — The data revealed maximum difference between CSP and Qβ-CSP immunogenicity was at the lowest antigen dose, particular for the NANP titers. (TIF) [file pone.0142035.s001.tif]

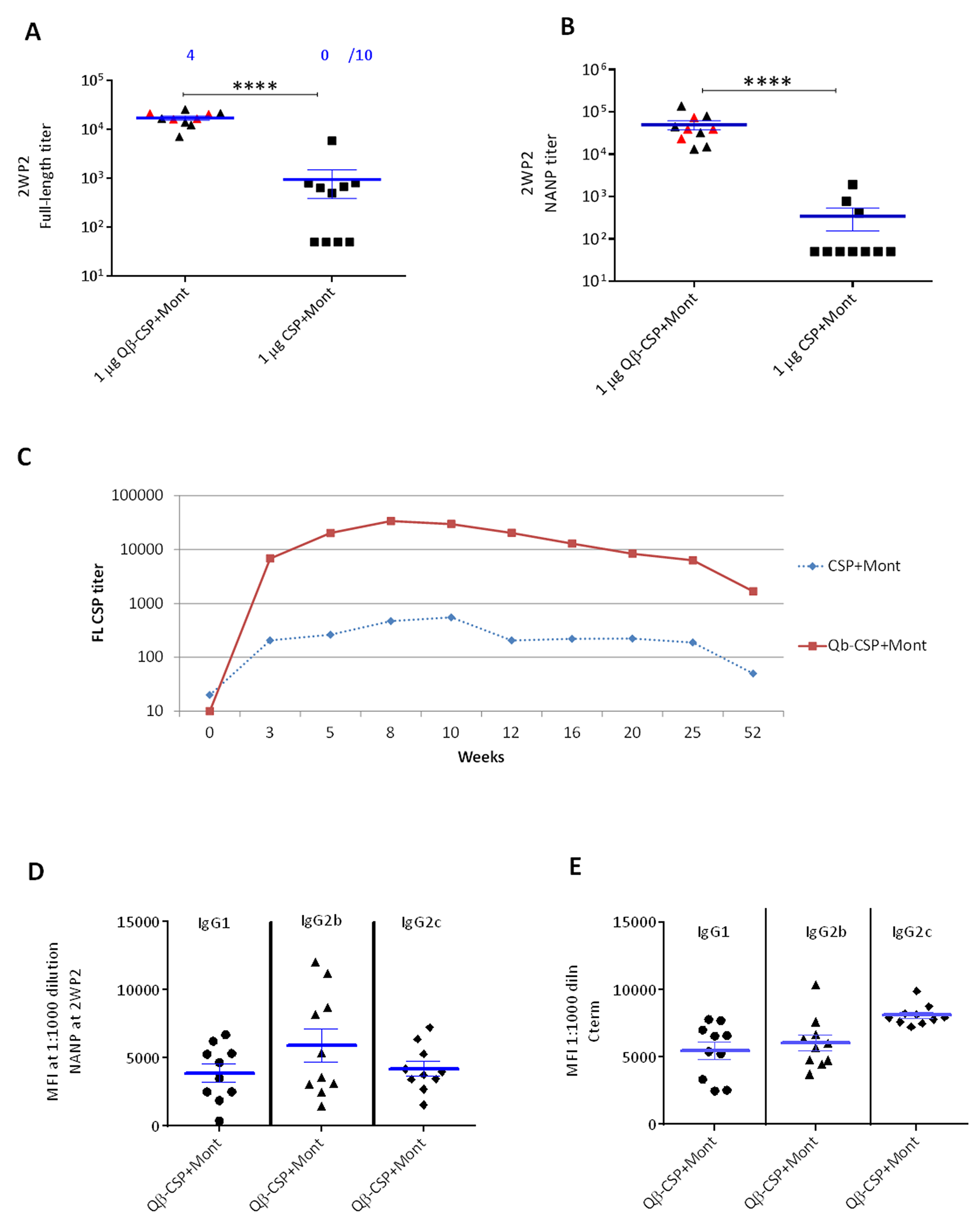

Supplement: S2 Fig — A, B show the FL and NANP response 2 weeks after the second vaccination of 10 challenged mice. **** (p<0.0001); red symbols represent protected mice and numbers protected out of 10 (blue). C, Mean full-length ELISA titer of 5 mice followed for 1 year. D, E show IgG1, IgG2b and IgG2c responses measured by Luminex and expressed as MFI at 1:1000 dilution against NANP peptide (D) or C-term protein (E). (TIF) [file pone.0142035.s002.tif]
